# Characteristics of Health Care Practices and Systems That Excel in Hypertension Control

**DOI:** 10.5888/pcd15.170497

**Published:** 2018-06-07

**Authors:** An Young, Matthew D. Ritchey, Mary G. George, Judy Hannan, Janet Wright

**Affiliations:** 1 Division of Preventive Medicine, Emory University, Atlanta, Georgia; 2 Division for Heart Disease and Stroke Prevention, National Center for Chronic Disease Prevention and Health Promotion, Centers for Disease Control and Prevention, Atlanta, Georgia

## Abstract

Approximately 1 in 3 US adults has hypertension, but only half have their blood pressure controlled. We identified characteristics of health care practices and systems (hereinafter practices) effective in achieving control rates at or above 70% by using data collected via applications submitted from April through June 2017 for consideration in the Million Hearts Hypertension Control Challenge. We included 96 practices serving 635,000 patients with hypertension across 34 US states in the analysis. Mean hypertension control rate was 77.1%; 27.1% of practices had a control rate of 80% or greater. Although many practices served large populations with multiple risk factors for uncontrolled hypertension, high control rates were achieved with implementation of evidenced-based strategies.

## Objective

Approximately 1 in 3 US adults (about 75 million) has hypertension, a major risk factor for heart disease and stroke, but the condition is controlled in only around half (1). Million Hearts, a national initiative with a 5-year goal of preventing 1 million cardiovascular events, includes an intense focus on increasing blood pressure (BP) control via multiple avenues including increased use of evidence-based clinical interventions (1,2). We identified common characteristics of practices and health systems (hereinafter practices) with exemplary BP control rates by examining practices that applied for recognition via the 2017 Million Hearts Hypertension Control Challenge.

## Methods

We collected data from applications (Appendix) to the Million Hearts Hypertension Control Challenge submitted from April through June 2017 (3). Practices were eligible to apply if they were US-based with a patient population of 500 or more and a BP control rate at or greater than 70% among patients aged 18 to 85 with diagnosed hypertension during a 12-month reporting period starting no earlier than January 1, 2016. Hypertension control was defined as BP below 140/90 mm Hg, per national guidelines (4). The control rate calculations performed by the practices conformed to National Quality Forum’s 0018 measure specifications (5). We collected information on the percentage of the practice’s patient population who were from racial/ethnic minority groups (as defined by the applicant), were uninsured or on Medicaid, or spoke a primary language other than English; the practice’s service area type (urban, rural, or both); total number of patients served who were aged 18 to 85 and the number of those patients with hypertension; BP control rates for 2015 and 2016; and strategies used to manage hypertension.

Practices were dichotomized on the basis of the population served into 2 groups, disparate and nondisparate. Disparate practices were those designated Federally Qualified Health Centers (N = 38), which serve mainly underserved areas or populations; practices with more than 50% of patients of racial/ethnic minority groups, uninsured, or on Medicaid; or practices with more than 50% of patients who spoke a primary language other than English (N = 10). Nondisparate practices were all others (N = 48). Differences were determined by using Open Epi version 3.01 (Open Source Epidemiologic Statistics for Public Health, www.openepi.com) to calculate 2-tailed χ^2^ or Fisher exact tests for categorical variables and Student’s *t* tests for continuous variables with significance set at *P* < .05.

## Results

We included 96 practices in our analysis from 34 states serving 2.45 million patients annually. Practices had a mean population of 25,535 (standard deviation [SD], 72,634) per practice, (Table 1). On average, 31.8% (SD, 28.7) of each practice’s population were minority patients; 27.6% (SD, 24.9) had Medicaid coverage. Half of the practices (N = 48) served disparate populations, and 59.4% had urban-only service areas. Almost all (N = 95, 98.9%) had electronic health record (EHR) systems.

**Table 1:** Characteristics of US Health Care Practices and Systems That Excel in Hypertension Control, by Patient Socioeconomic Characteristics, 2016^a^

Practice and System Characteristic	All Practices and Systems (N = 96)	Practices and Systems Serving Disparate Populations^b ^(N = 48)	Practices and Systems Serving Nondisparate Populations^c^ (N = 48)	*P* Value
**Total patients aged 18–85 y seen at least once during reporting period^a^, millions**	2.45	0.89	1.56	NA
**No. of patients, mean (SD)**	25,535 (72,634)	18,583 (31668)	32,487 (97,786)	<.001^d^
**Age, y, N millions (%)**				
18–44	1.07 (43.5)	0.45 (50.0)	0.62 (39.8)	<.001^e^^,^ f
45–64	0.89 (36.2)	0.31 (35.1)	0.57 (36.8)	
65–74	0.31 (12.6)	0.08 (9.4)	0.22 (14.4)	
75–85	0.18 (7.3)	0.05 (5.3)	0.13 (8.4)	
**Patients with disparities, mean percentage (SD)**				
Minority	31.8 (28.7)	46.0 (32.2)	17.6 (14.6)	<.001^d^
Medicaid	27.6 (24.9)	44.4 (22.3)	10.9 (13.5)	<.001^d^
Non-English as primary language	16.4 (23.3)	27.0 (28.1)	5.8 (8.8)	<.001^d^
Uninsured	11.0 (14.3)	17.0 (16.8)	5.0 (7.7)	<.001^d^
**Service area, N (%)**				
Urban	57 (59.4)	23 (47.9)	34 (70.8)	.048^e^^,^ g
Rural	24 (25.0)	14 (29.2)	10 (20.8)	
Both urban and rural	15 (15.6)	11 (22.9)	4 (8.3)	
**Patients with diagnosed hypertension**				
Total, N	635,255	222,051	413,204	NA
Prevalence, mean percentage (SD)	32.5 (21.0)	30.7 (23.0)	34.4 (18.8)	.39^d^
Control rate, mean percentage (SD)	77.1 (6.3)	76.1 (5.7)	78.1 (6.8)	.12^d^
Control rate ≥80%, N (%)	26 (27.1)	11 (22.9)	15 (31.2)	.49^h^
**Change in rate of hypertension control from 2015 through 2016, N (%)**				
Increase	65 (67.7)	35 (72.9)	30 (62.5)	.36^h^^,^ i
Decrease	27 (28.1)	11 (22.9)	16 (33.3)	NA
Missing 2015 rate or no change in rate	4 (4.2)	2 (4.2)	2 (4.2)	NA
Absolute change in rate, mean (SD)	2.2 (5.3)	2.6 (4.5)	1.7 (5.9)	.40^d^

Abbreviations: NA, not applicable; SD, standard deviation.

a Data were collected from applications to the Million Hearts Hypertension Control Challenge submitted from April through June 2017. Practices and systems were eligible to apply if they had a blood pressure control rate at or greater than 70% among patients aged 18 to 85 with diagnosed hypertension during a 12-month reporting period starting no earlier than January 1, 2016; 86 (89.6%) of the 96 practices and systems had a reporting period from January 1, 2016 to December 31, 2016.

b We defined disparate patients are those served in a Federally Qualified Health Center or a practice or system in which more than 50% of the patient population is either a racial/ethnic minority (as determined by applicant), uninsured, on Medicaid, or speaks a non-English primary language.

c We defined nondisparate patients are those served in a practice or system that is not a Federally Qualified Health Center or in which ≤50% of patient population is either a racial/ethnic minority, uninsured, on Medicaid, or speaks a non-English primary language.

d Calculated by using a *t* test.

e Calculated by using a χ^2^ test.

f Degrees of freedom = 3.

g Degrees of freedom = 2.

h Calculated by using a Fisher exact test.

i Excludes practices and systems that could not provide a 2015 hypertension control rate or that had no change in the rate.

Mean hypertension prevalence was 32.5% (SD, 21.0) and the mean BP control rate was 77.1% (SD, 6.3) (Table 1); 27.1% reported control rates of 80% or more, thereby meeting the goal newly established by Million Hearts 2022 (6). Most (n = 65, 67.7%) reported a BP control rate increase in 2016 compared with 2015 (mean, 2.2 percentage point increase; SD, 5.3).

Compared with practices serving nondisparate patient populations, practices serving disparate populations tended to more often have rural or combined urban/rural service areas, and, despite treating a younger population, had similar hypertension prevalence (Table 1). Practices’ mean BP control rates were similar when stratified by disparity status.

Various strategies were implemented to achieve BP control rates at or above 70% (Table 2). Compared with practices serving nondisparate populations, practices serving disparate populations reported greater use of a combination of EHR features, treatment protocols, care coordinator involvement on BP management teams, and patient outreach strategies.

**Table 2:** Hypertension Control Strategies Implemented by US Health Care Practices and Systems That Excel in Hypertension Control, by Patient Socioeconomic Status, 2016^a^

Strategy	All Practices and Systems^b^ (N = 96)	Practices and Systems Serving Disparate Populations^c^ (N = 48)	Practices and Systems Serving Nondisparate Populations^d^ (N = 48)	*P* Value^e^
**Electronic health record features, N (%)**				
Electronic prescribing	87 (90.6)	48 (100.0)	39 (81.3)	.003
Patient summary reports **f**	87 (90.6)	42 (87.5)	46 (95.8)	.27
Patient registry **g**	65 (67.7)	36 (75.0)	29 (60.4)	.19
Clinical decision supports **h**	65 (67.7)	42 (87.5)	23 (47.9)	<.001
Treatment/testing reminders^i^	62 (64.6)	35 (72.9)	27 (56.3)	.13
Provider dashboard **j**	56 (58.3)	28 (58.3)	28 (58.3)	.99
≥5 Features	46 (47.9)	29 (60.4)	17 (35.4)	.02
**Written treatment protocol N, (%)**	49 (51.0)	34 (70.8)	15 (31.3)	<.001
**Team-based care N, (%)**				
Nurse engagement	65 (67.7)	36 (75.0)	29 (60.4)	.19
Care coordinator engagement	60 (62.5)	37 (77.0)	24 (50.0)	.01
Nurse practitioner engagement	49 (51.0)	29 (60.4)	20 (41.7)	.10
Clinical pharmacist engagement	30 (31.3)	17 (35.4)	13 (27.0)	.51
Behavioral health engagement	5 (5.2)	5 (10.4)	0 (0.0)	.06
≥3 personnel above involved	39 (40.6)	24 (50.0)	15 (31.3)	.10
**Incentives N, (%)**				
Provider, financial^k^	25 (26.0)	15 (31.3)	10 (20.8)	.35
Provider recognition^l^	24 (25.0)	10 (20.8)	15 (31.3)	.35
Provider, administrative time^m^	5 (5.2)	3 (6.3)	2 (4.2)	.99
Patient (eg, insurance gift cards, sliding scale fee)	4 (4.2)	2 (4.2)	1 (2.1)	.99
**Patient engagement N, (%)**				
Free blood pressure check clinics	56 (58.3)	32 (66.7)	24 (50.0)	.15
Patient outreach^n^	50 (52.1)	32 (66.7)	18 (37.5)	.008
Medication adherence strategies^o^	43 (44.8)	22 (45.8)	21 (43.8)	.99
Home blood pressure monitoring	38 (39.6)	23 (47.9)	15 (31.3)	.14

a Data were collected from applications to the Million Hearts Hypertension Control Challenge submitted from April through June 2017. Practices and systems were eligible to apply if they had a blood pressure control rate at or greater than 70% among patients aged 18 to 85 with diagnosed hypertension during a 12-month reporting period starting no earlier than January 1, 2016.

b Some practices and systems reported multiple strategies; therefore, percentages do not total 100%.

c We defined disparate patient populations are those served in a Federally Qualified Health Center or a practice or system in which more than 50% of the patient population is either a racial/ethnic minority (as determined by applicant), uninsured, on Medicaid, or speaks a non-English primary language.

d We defined nondisparate patient populations are those served in a practice or system that is not a Federally Qualified Health Center or in which ≤50% of patient population is either a racial/ethnic minority (as determined by applicant), uninsured, on Medicaid, or speaks a non-English primary language.

e Calculated by using a Fisher exact test.

f Based on summary information about the patient’s most recent blood pressure readings and hypertension-related interventions that inform health care provider’s point-of-care decision making.

g A collection of hypertension-related information about a group of patients used for quality improvement by practices and systems.

h Automated methods, often using electronic health record systems, to identify circumstances in which health care providers identify actions to reinforce use of evidence-based interventions with patients.

i Tools that prompt health care to follow-up with patients within a prescribed timeframe.

j An overview of how a provider or system’s entire patient panel is doing in blood pressure control.

k Payment for achieving blood pressure control among patients (eg, receipt of a quarterly or annual bonus).

l Publication of provider’s performance compared with other providers in a practice or system; recognition based on quality metrics (eg, being able to reach certain blood pressure control goals).

m Provider given time away from patients to plan and organize blood pressure management efforts.

n Contacting patients (eg, by mail or telephone) who do not have regular follow-up in clinic; medical staff organizing and attending community health fairs to screen patients for hypertension; care coordinators working with emergency departments and hospitals to follow up on patients with recent hospital visits.

o Having pharmacist onsite to answer questions, involving family members to improve compliance, medication reconciliation at each visit, having case management nurses contact patients to verify understanding and adherence to treatment plans, assessing barriers to medication adherence, providing a 3-month supply of medication with refills, refill reminders or automatic refills.

## Discussion

About 90% of US adults with uncontrolled hypertension reported having a usual source of health care (1). Our study showed that high rates of BP control (ie, ≥70%) can be achieved among adults with hypertension who have access to health care by using multifaceted, system-wide approaches. Moreover, our study shows that high BP control rates are achievable even among patient populations at high risk for having uncontrolled BP, including certain minority groups (eg, African Americans, Hispanics), the uninsured, and those who are socioeconomically disadvantaged (7).

Every practice included in our analysis reported using multiple strategies to achieve high BP control rates. Use of these strategies aligns with the World Health Organization’s Innovative Care for Chronic Conditions recommendations to improve outcomes and reduce disparities in hypertension control (8) and with the hypertension management strategies recommended by the Centers for Disease Control and Prevention (CDC)-funded State Public Health Actions grantees and Million Hearts (1,6,9). Almost every practice reported having EHRs, and most used EHR features such as electronic prescribing, patient registries, and clinical decision support tools to better track and manage their patients with hypertension (10). In addition, over half reported implementing hypertension treatment protocols. Protocol use helps standardize and coordinate care and facilitates a team approach to BP management that leverages the skills and reach of multiple types of health care professionals to maximize BP control (1,11,12). Moreover, some practices provided financial and other incentives to clinicians and patients to encourage greater attention to BP control (1). Finally, many practices engaged patients in BP home monitoring to assess progress, inform decision making, and encourage adherence to treatment regimens (12).

Compared with practices serving nondisparate populations, those serving disparate populations reported relying on various types of personnel on patient care teams to achieve hypertension control. For example, practices serving disparate populations were more likely to include care coordinators and behavioral specialists who can address potential barriers to care, such as financial issues, transportation, and mental health issues. Moreover, these practices were more likely to use standardized treatment protocols and proactive outreach strategies to engage with patients outside of the clinic.

Our study had limitations. First, the data are self-reported, and only the top 30% of practices underwent data verification during the Million Hearts Hypertension Control Challenge process. Second, strategies implemented by practices included in our study may not be generalizable to other high-performing practices because ours was not a random sample. Third, information was not collected among practices that were not high performing to determine whether BP control rates differed by strategies used. Finally, because multiple comparisons were performed without adjustment, results should be interpreted with caution. Although we cannot attribute improvement in BP control to specific strategies, our findings show that with implementation of evidence-based, multidisciplinary, system-wide strategies, high BP control rates are achievable in diverse types of practices and collectively at the population level.

## Appendix. Million Hearts Hypertension Control Champion Nomination

This file is available for download as a Microsoft Word document.
