# Supplementary material for: Characteristics of Health Care Practices and Systems That Excel in Hypertension Control
Source: Prev Chronic Dis. 2018 Jun 7;15:E73. doi: 10.5888/pcd15.170497 (PMC6016404; doi:10.5888/pcd15.170497)
Supplement: Supplementary file 1 [file 17_0497Appendix.docx]

**Appendix**

**Million Hearts® Hypertension Control Champion Nomination**

Public reporting burden of this collection of information is estimated at 30 minutes per response, including the time for reviewing instructions, searching existing data sources, gathering and maintaining the data needed, and completing and reviewing the collection of information. An agency may not conduct or sponsor, and a person is not required to respond to, a collection of information unless it displays a currently valid OMB control number. Send comments regarding this burden estimate or any other aspect of this collection of information, including suggestions for reducing this burden to CDC/ATSDR Information Collection Review Office, 1600 Clifton Road, NE, MS D-74, Atlanta, GA 30333, ATTN: PRA 0920-0976.

**Contact information (for individual submitting the nomination):**

Name: ________________________________________________________________________

Business Address: _______________________________________________________________

City: ______________________ State: __________ Zip Code: ______________

Business Phone:____________________ Business E-mail: _______________________________

Check the box which represents your relationship with the nominee:

- I am the nominee
- Employee of Nominee
- Contract with Nominee
- State health department
- Other ______________

**Nominee information: Please provide the following information for the provider or practice being entered into the Challenge. Nominate either practice or provider, but not both.**

Practice Name (if the practice is the nominee): ________________________________________________________________________

Provider Name (if the provider is the nominee):

________________________________________________________________________

Business Address: ______________________________________________________________

Business Phone: _________________ Business E-mail: ____________________________

Check the box which best represents the nominee:

- A healthcare system
- A single clinician or group practice or clinic

Check the box which best represents the nominee’s practice:

- Obstetrics/gynecology
- Family practice
- Internal medicine
- Osteopathy
- Cardiovascular care
- Other ________________________

**Population served**

Number of patients enrolled in the practice or health system: _____________________

Describe patient demographics that support the practice or health system’s care for a population with a high prevalence of hypertension:

Geographic location of clinic (select both if you are a health system and both apply): 🞏 Rural 🞏 Urban
Percent of patients who belong to a racial/ethnic minority: ________________________

Percent of patients whose primary language is not English: ________________________

Percent of patients who are enrolled in Medicaid: ________________________

Percent of patients who have no health insurance: ________________________

Other ___________________________________________________________________

**Hypertension Control**

Nominees are asked to provide two hypertension control rates: a current rate for a 12-month period and a previous rate for a 12 month period a year or more before.

CDC supports the definition of “hypertension control” as patients aged 18 through 85 years who had a diagnosis of hypertension and whose blood pressure was adequately controlled ( <140 mmHg systolic and <90 mmHg diastolic).

For the current Hypertension Control Rate:

What is the reporting period (e.g., 1/1/2015 to 12/31/2015)? ________________.

For the current reporting period, the nominee used which of the following clinical quality measure to define hypertension control. Please check the appropriate box below and provide the requested information:

- National Quality Forum (NQF) 0018 guidelines Describe the exclusions the nominee includes (e.g., pregnant women, patients with end-stage renal disease). __________________________________________________________________________
- CMS Physician Quality Reporting System (PQRS) 236 guidelines. Describe the exclusions the nominee includes (e.g., pregnant women, patients with end-stage renal disease).______________________________________________________________
- CMS 165v3 guidelines. Describe the exclusions the nominee includes (e.g., pregnant women, patients with end-stage renal disease).___________________________________________________________________
- NCQA HealthCare Effectiveness Information Set (HEDIS). Describe the exclusions the nominee includes (e.g., pregnant women, patients with end-stage renal disease).____________________________________________________________________
- HRSA Uniform Data System (UDS). Describe the exclusions the nominee includes (e.g., pregnant women, patients with end-stage renal disease).__________________________________________________________________
- Other. Describe how the nominee calculates the measure; including who is included in the denominator and what is considered adequate control.

______________________________________________________________________________
______________________________________________________________________________
______________________________________________________________________________

**Calculation of Hypertension Control Rate**

Of the number of number of patients enrolled in the practice or health system, how many adult patients (18 – 85 years old) were seen at least once during the reporting period? ______________________

Of this number of patients seen, distribute them by age:

Percent of patients: Age: 18-44 _______

Percent of patients: Age 45-64: _______

Percent of patients: Age 65-74: ________

Percent of patients: Age 75-85: _______

Of the number of adult patients (18-85 years old) seen during the reporting period, how many were diagnosed with hypertension? ________

Of the number of adult patients (18-85 years old) diagnosed with hypertension, how many are included in the control rate denominator (after removing the exclusions listed above; e.g., pregnant women, patients with end-stage renal disease)? _____________

How many of the patients were excluded from the denominator?___________

How many of the patients in the denominator had their blood pressure in control (numerator)?___________

What was the Hypertension Control Rate for the practice or healthcare system’s adult hypertensive population during this reporting period (numerator/denominator)? __________________

**For the previous period Hypertension Control Rate:**

For the previous reporting period, did the nominee use the same clinical quality measure guidelines as the current reporting period?

- Yes.
- No.

If not, which clinical quality measure guideline was used?______________________________

Using the same steps, what was the Hypertension Control Rate for the practice or healthcare system’s adult hypertensive population during previous reporting period? ______________

What was the previous reporting period (e.g., 1/1/2014 to 12/31/2014): ___________

**Additional Hypertension Control Rate Questions**

Do you report hypertension control rate to any other federal or regulatory agency?

- Yes. Which one? _________________
- No.

Were the data obtained from an electronic health record system? ________.

If not, how were the data obtained? ________________________________________________

**Clinical system supports**

Please check the button before each sustainable process for providing care in the clinic or healthcare system that is used on a regular basis. Provide a brief description of as many “other” processes or systems as applicable to your practice or health system. You may also add details to many of the systems described below to support the nomination.

- Written treatment protocols
- Electronic Medical Records (EMR): Registry features
- Electronic Medical Records (EMR): With clinical decision supports
- Electronic Medical Records (EMR): With e-prescribing
- Electronic Medical Records (EMR): With treatment/testing reminders
- Electronic Medical Records (EMR): With patient summary reports
- Team Based Care: Nurse engagement
- Team Based Care: Nurse Practitioner engagement
- Team Based Care: Pharmacist engagement
- Team Based Care: Patient Navigator/Care Coordinator
- Team Based Care: Other

Please describe: ______________________________________________________________
___________________________________________________________________________

- Provider Incentives: Financial

Please describe: ______________________________________________________________
___________________________________________________________________________

- Provider Incentives: Administrative

Please describe: ______________________________________________________________
___________________________________________________________________________

- Provider Incentives: Recognition

Please describe: ______________________________________________________________
___________________________________________________________________________

- Provider Incentives: Other

Please describe: ______________________________________________________________
___________________________________________________________________________

- Patient Incentives

Please describe: ______________________________________________________________
___________________________________________________________________________

- Non-electronic reminders or alerts for providers or patients
- Free blood pressure checks
- Provider Dashboards

Please describe: ______________________________________________________________
_________________________________________________________________________

- Home blood pressure monitoring support or equipment

Please describe: ______________________________________________________________
_________________________________________________________________________

- Medication adherence strategies

Please describe: ______________________________________________________________
___________________________________________________________________________

- Outreach to patients

Please describe: ______________________________________________________________
___________________________________________________________________________

- Other

Please describe: ______________________________________________________________
___________________________________________________________________________

Is there anything else you would like to add to support the nomination?
_______________________________________________________________________________

_______________________________________________________________________________

_______________________________________________________________________________

**Agreement to Participate**

Please enter your name below to indicate that you, as the nominee, agree to the following:

If you are not the nominee, please enter your name below assuring that you have consulted with the nominee, and the nominee agrees to the following:

- All information provided is true and accurate to the best of your knowledge.
- To participate in a data verification process if selected as a candidate for champion.
- Consent to a background check if selected as a candidate for champion.
- To be recognized by provider or practice name and location if selected as a champion, to participate in recognition activities, and to share best practices for the development of publically available resources.
- To assume any and all risks and waive claims against the Federal Government and its related entities, except in the case of willful misconduct, for any injury, death, damage, or loss of property, revenue, or profits, whether direct, indirect, or consequential, arising from my participation in this prize contest, whether the injury, death, damage, or loss arises through negligence or otherwise.
- To indemnify the Federal Government against third party claims for damages arising from or related to competition activities.”

___________________________________________________________________

Submit Nomination

Thank you for participating.
